# Single Laboratory Validation of a Quantitative Core Shell-Based LC Separation for the Evaluation of Silymarin Variability and Associated Antioxidant Activity of Pakistani Ecotypes of Milk Thistle (*Silybum Marianum* L.)

**DOI:** 10.3390/molecules23040904

**Published:** 2018-04-14

**Authors:** Samantha Drouet, Bilal Haider Abbasi, Annie Falguières, Waqar Ahmad, Sumaira, Clothilde Ferroud, Joël Doussot, Jean Raymond Vanier, Eric Lainé, Christophe Hano

**Affiliations:** 1 Laboratoire de Biologie des Ligneux et des Grandes Cultures (LBLGC), INRA USC1328, Université d’Orléans, Pôle Universitaire d’Eure et Loir, 21 rue de Loigny la Bataille, 28000 Chartres, France; samantha.drouet@univ-orleans.fr (S.D.); bhabbasi@qau.edu.pk (B.H.A.); joel.doussot@cnam.fr (J.D.); eric.laine@univ-orleans.fr (E.L.); 2 Bioactifs et Cosmétiques, GDR 3711 COSMACTIFS, CNRS/Université d'Orléans, 45100 Orléans, France; 3 Department of Biotechnology, Quaid-i-Azam University, Islamabad 45320, Pakistan; awaqar@bs.qau.edu.pk (W.A.); sumaira.khan1890@gmail.com (S.K.); 4 Ecole Sciences industrielles et technologiques de l’information (SITI), Département Chimie Alimentation Santé Environnement Risque (CASER), Le CNAM 75141 Paris Cedex 03, France; annie.falguieres@cnam.fr (A.F.); clotilde.ferroud@lecnam.net (C.F.); 5 Plantes Médicinales et Aromatiques 28, PMA28, 1, place de l’Eglise, 28140 Varize, France; jraymond.vanier@pma28.fr

**Keywords:** antioxidant activities, core shell column, ecotypes, LC-MS, *Silybum marianum*, silymarin

## Abstract

Fruits of *Silybum marianum* (L.) Gaernt are the main source of taxifolin derived flavonolignans. Together, these molecules constitute a mixture called silymarin with many useful applications for cosmetic and pharmaceutic industries. Here, a validated method for the separation of the silymarin constituents has been developed to ensure precision and accuracy in their quantification. Each compound was separated with a high reproducibility. Precision and repeatability of the quantification method were validated according to the AOAC recommendations. The method was then applied to study the natural variability of wild accessions of *S. marianum*. Analysis of the variation in the fruits composition of these 12 accessions from Pakistan evidenced a huge natural diversity. Correlation analysis suggested a synergistic action of the different flavonolignans to reach the maximal antioxidant activity, as determined by cupric ion reducing antioxidant capacity (CUPRAC) and ferric reducing antioxidant power (FRAP) assays. Principal component analysis (PCA) separated the 12 accessions into three distinct groups that were differing from their silymarin contents, whereas hierarchical clustering analysis (HCA) evidenced strong variations in their silymarin composition, leading to the identification of new silybin-rich chemotypes. These results proved that the present method allows for an efficient separation and quantification of the main flavonolignans with potent antioxidant activities.

## 1. Introduction

**Figure 1:**
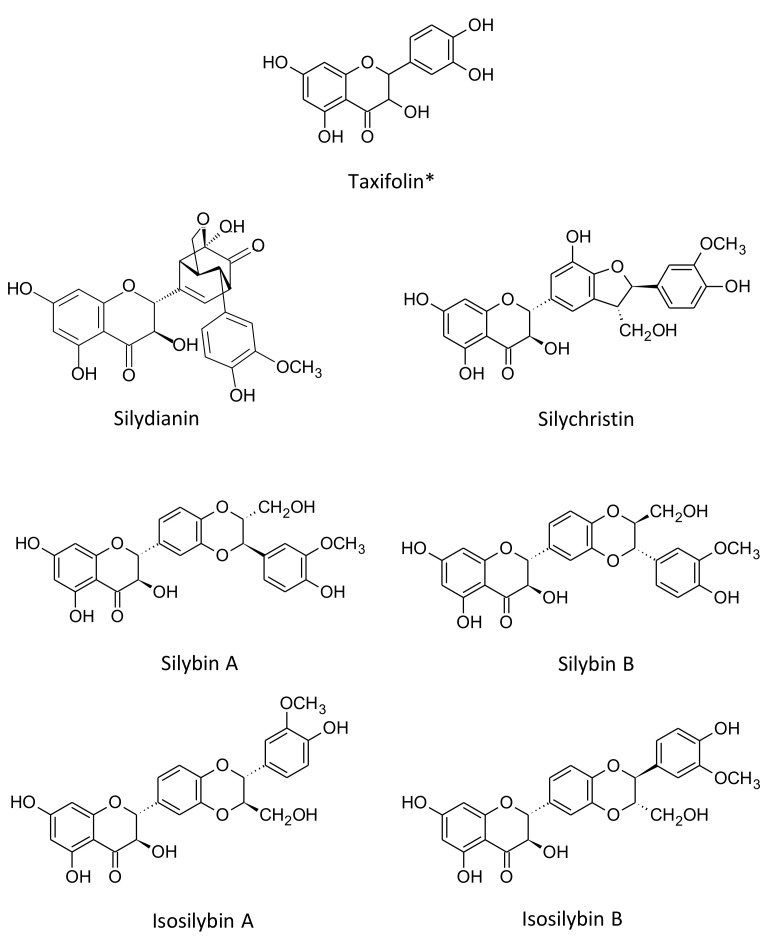
Chemical structure of the main six flavonolignans and one flavonoid (*) constituting the Silymarin mixture from *Silybum marianum* achene extract.

The fruit of milk thistle (*Silybum marianum* L.) is a rich source of phytochemicals compounds with multiple biological interest. Among them, silymarin is accumulated in the pericarp (the most external part of the fruit) up to 9% (*w*/*w*) and it is composed of a mixture of flavonolignans: silybin A, silybin B, silydianin, silychristin, isosilybin A, and isosilybin B, deriving from taxifolin their common flavone precursor (Figure 1) [1,2,3,4,5,6,7].

Milk thistle has been used as a medicinal plant for centuries as a remedy for various diseases [8,9]. Its extract is used as food supplements based on its traditional use in the European pharmacopoeia as liver detoxifier [10] and as an antidote against *Amanita phalloides* intoxication [11,12]. Those effects have been related to its well-known hepatoprotective action [2,3,4,13,14], limiting acute liver injuries thanks to pronounced anti-inflammatory actions and antioxidant activity [15], which is described at both the cellular and molecular levels [16]. Its main constituent Silybin have been largely studied and proposed to display various health promoting effects, such as hypocholesterolaemic action [17] or potential chemopreventive action in human prostate carcinoma [18]. Topical applications of silybin also display a large array of beneficial actions on skin: anti-inflammatory [19], protection against UV-B radiations and sun burns [20,21,22], antiglycation action [23], and prevention of skin cancers [24]. 

All of those biological activities evidence the great potential of *Silybum marianum* extracts for pharmaceutical and cosmeceutical applications. Unfortunately, as reported by Chambers et al. [25], in most of the studies dealing with biological activities of *S. marianum* extract, their composition is rarely determined. Since silymarin content and composition greatly vary in large amounts depending on the extraction procedure, the genetic background, and/or the edaphic parameters, this could result in reproducibility and validity issues [25]. From a chemical point of view, silymarin is composed of different isomers that are difficult to separate. This could explain why, in many cases, publications that are dealing with the elucidation of *S. marianum* biological activities, silymarin is usually quantified as a whole whereas the relative amounts of the different constituents can vary and are not measured. Among these different constituents, silybins are the major bioactive component of *S. marianum* extract [6,7], thus reinforcing the need of validated (quantitative) methods for the accurate evaluation of their concentration levels.

The understanding of the flavonolignans biosynthesis is currently an important factor to progress in the use of these molecules. Learning more about the natural genetic influencing flavonolignans accumulation is an important step. Native from the Mediterranean area, *S. marianum* propagates through the world with a vigorous growth in warm and dry regions [2,26]. It was observed to develop locally adapted subpopulations, which are also known as ecotypes, which can differ by their phenotype, growth, tolerance for temperature, light, nutrients, and/or stress [27]. These ecotypes also displayed a wide range of variation in their silymarin content and composition, as described for ecotypes from Egypt, Iran, Poland, Hungary, Bulgaria, Germany, Canada, and New Zealand [25,28,29,30,31,32]. Because of the very rapid propagation of this plant, milk thistle has become a widespread weed, especially in north Pakistan, being widely used by phytomedicine industry [32]. However, variability in silymarin concentration and composition of wild *S. marianum* accessions from Pakistan have never been studied. Investigating the content of various ecotypes would help to discover the best plants for phytomedicine and cosmetic applications. In this context, optimizing the accurate and quantitative separation protocol for these compounds in a simple way is essential to facilitate their further exploration. For this purpose, we have first developed and validated a quantitative and reproducible LC-based method for the separation of the different flavonolignans from *S. marianum* fruits using core-shell technology. Subsequently, this method was applied to investigate the natural variability in the silymarin content of wild populations of *S. marianum,* which are grown in Pakistan in order to enhance our current understanding of the chemical diversity and to estimate associated antioxidant capacities of these compounds.

## 2. Materials and Methods 

### 2.1. Plant Material

The achenes of *Silybum marianum* of Pakistani purple ecotypes were collected by the Department of Biotechnology from Quaid-i-Azam University and identified by Dr BH Abbasi [32].

### 2.2. Chemicals

All of the solvents and reagents for extraction and HPLC analysis were of analytical grade or the highest available purity (Thermo Fischer Scientific, Illkirch, France). Deionized water was purified by a Milli-Q water-purification system (Merck Millipore Fontenay sous Bois, Paris, France). All of the solutions that were prepared for HPLC were filtered through 0.45 µm nylon syringe membranes prior to use. Silybin A-B (1612630), silybin B (95684), isosilybin A (97326), isosilybin B (59527), silydianin (30494), and silychristin (51681) standards were purchased from Sigma Aldrich (Saint-Quentin Fallavier, France). Standardized commercial silymarin mixture (S0292) was purchased from Sigma Aldrich.

### 2.3. Extraction

Samples of 60mg milled inflorescences dry weight (DW) were extracted in 1 mL of 50% ethanol solvent. The ultrasonic bath used was a USC1200TH (VWR International, Fontenay-sous-Bois, France; inner dimension: 300 mm × 240 mm × 200 mm). The extraction was conducted for 60 minutes at an ultrasound frequency of 45 kHz and a temperature of 25 °C. According to Corbin et al., 2015 [33], the extract supernatant was filtered through 0.45 µm nylon syringe membranes before HPLC injection. 

### 2.4. HPLC-ESI-MS Analysis

Silymarin compounds were quantified using a LC-MS analysis that was performed on a Water 2695 Alliance (Waters-Micromass, Manchester, UK) coupled with a single quadrupole mass spectrometer ZQ (Waters-Micromass, Manchester, UK). LC-ESI-MS data were collected in the positive and negative modes. Data acquisition and processing were performed with MassLynx 4.0 software (Waters-Micromass, Manchester, UK). The separation was performed at 35 °C on a core-shell column (Kinetex 5 µm XB-C18, 100 Å, LC Column 150 × 4.6 mm, C18 with iso-butyl side chains, and with TMS endcapping, core-shell silica, Phenomenex Le Pecq France). The mobile phase was composed with a mixture of methanol (solvent A) and HPLC grade water (solvent B), both being acidified with 0.05% formic acid. A linear gradient was applied for the mobile phase variation, ranging from a 5:95 (*v*/*v*) to 100:0 (*v*/*v*) mixture of solvents A and B, respectively, with a flow rate of 1.30 mL/min. The injection volume was 3 µL, the maximum back pressure was 110 bar and the detection was performed at 280 nm. Flavonolignans were identified by comparison with authentic standards (Sigma Aldrich). The limits of detection (LOD) and the limits of quantification (LOQ) were determined based on the signal-to-noise ratio (S:N) of 3:1 and 10:1, respectively.

### 2.5. Method Validation

The separation method was validated as follows: The linear correlations between peak area and standard concentrations were found to be high in the range of 0.5–50 µg·mL^−1^. The resulting linear equations were obtained with R^2^-value for a six-point calibration graph >0.99, and the slopes of five replicates of the calibration graph covering the analytical range for each standard varied no more than 1% in terms of RSD over a period of four weeks. The LOD and LOQ were calculated using S/N ratio of 3 and 10, respectively. The repeatability precision was evaluated by six injections of the same sample that were performed on the same day. Stability was evaluated by calculating the recovery, RSD and average recovery using spiked concentration additions at three different levels (25, 50, and 100 ppm). The matrix effect was determined by calculating the recovery from two different matrixes (commercial silymarin preparation (Sigma Aldrich) and non-defatted whole fruit extract, which was prepared as described in paragraph 2.3).

Uncertainty estimation of the final quantitative results was evaluated using standard uncertainty calculations for intra-laboratory analytical procedure devoted to natural antioxidants deriving from plant materials [34,35,36]. Uncertainties $u_{\mathrm{standard}}$ resulting from stock and calibration standard preparation, $u_{\mathrm{purity}}$ resulting from standard purity, $u_{\mathrm{recovery}}$ and $u_{\mathrm{precision}}$ resulting from analytical method recovery and precision characteristics were calculated, as described by Sin et al (2006) [34].

$$u_{\mathrm{standard}}=\sqrt{{\left(u_{\mathrm{stock}}\right)}^{2}+{\left(u_{\mathrm{volume}}\right)}^{2}+{\left(u_{\mathrm{pipette}}\right)}^{2}}$$

uncertainty of standard preparation calculated using

$$u_{\mathrm{stock}}=\sqrt{{\left(u_{\mathrm{balance}}\right)}^{2}+{\left(u_{\mathrm{volume}}\right)}^{2}}$$

with uncertainty of analytical balance u_balance_ (Mettler Toledo, Viroflay, France) and uncertainty of volume u_volume_ for volumetric flasks (Thermo) and pipettes (Eppendorf, Montesson, France), which were calculated using manufacturers specifications.

u_purity_ calculated using purity percentages provided by manufacturer (Sigma Aldrich).

$$u_{\mathrm{recovery}}=\sqrt{\frac{{\mathrm{RSD}}_{1}^{2}\left(n_{1}-1\right){+\mathrm{RSD}}_{2}^{2}\left(n_{2}-1\right)}{\left(n_{1}-1\right)+\left(n_{2}-1\right)}}$$

calculated as the sum of the relative standard deviation (RSD) percentage of spike recovery (RSD_1_) and the difference from the standardized commercial preparation (S0292, Sigma Aldrich) (RSD_2_).

$$u_{\mathrm{precision}}=\frac{\mathrm{SD}}{\sqrt{n}}$$

calculated using the standard deviation (SD) of the relative percentage difference of repeatability precision results, where n corresponds to the number (6) of independent replicates.

$$u_{\mathrm{Combined}}=\sqrt{{\left(u_{\mathrm{standard}}\right)}^{2}+{\left(u_{\mathrm{purity}}\right)}^{2}+{\left(u_{\mathrm{recovery}}\right)}^{2}+{\left(u_{\mathrm{precision}}\right)}^{2}}$$

calculated as the square root of the sum of individual uncertainty.

### 2.6. Determination of Total Phenolic Production 

The total phenolic content (TPC) was analyzed by using Folin-Ciocalteu (FC, Sigma Aldrich) reagent, according to the method that was reported in [37]. 10 µL of milk thistle sample was mixed with 180 µL of a preparation composed to 4% Na_2_CO_3_ (in NaOH 0.1 M), 0.02% potassium sodium tartrate tetrahydrate and 0.02% CuSO_4_. After 10 min of incubation at room temperature (25 ± 2 °C), 10 µL of the FC reagent (diluted 1/3 with distilled water) were added to the above mixture and after 30 min of incubation at room temperature, the absorbance was measured at 735 nm with a spectrophotometer (BioTek ELX800 Absorbance Microplate Reader, BioTek Instruments, Colmar, France). Gallic acid (Sigma Aldrich) was used as the standard for the calibration curve, and the total phenolic contents (TPC) were expressed as milligrams gallic acid equivalents per gram.

Total phenolic production (mg·L^−1^) = DW (g·L^−1^) × TPC (mg·g^−1^)

### 2.7. Determination of Total Flavonoid Production 

Total flavonoid content (TFC) was determined by using aluminum chloride (AlCl_3_, Sigma Aldrich) colorimetric method that was reported by Xu et al., 2015 [38] with minor modifications. Briefly, 10 μL of the *Silybum marianum* sample was mixed with 10 μL of potassium acetate (1 M) and 10 μL of AlCl_3_ (10%, *w*/*v*) and 170 µL double distilled water. This mixture was incubated for 30 min at room temperature (25 ± 2 °C), and then the absorbance was measured at 415 nm by using UV–visible spectrophotometer (BioTek ELX800 Absorbance Microplate Reader, BioTek Instruments). Quercetin (Sigma Aldrich) was used as the standard for the calibration curve, and the total flavonoid contents were expressed as milligrams quercetin equivalents per gram. 

Total flavonoid production (mg·L^−1^) = DW (g·L^−1^) × TFC (mg·g^−1^)

### 2.8. Determination of Antioxidant Activity 

For antioxidant activity determination, ferric reducing antioxidant power (FRAP) was used by [39] and cupric ion reducing antioxidant capacity (CUPRAC) by [40] with little modification. Briefly, for FRAP 10 μL of the extracted sample was mixed with 190 μL of FRAP (10 mM TPTZ; 20 mM FeCl_3_, 6H_2_O and 300 mM acetate buffer pH3.6; ratio 1:1:10 (*v*/*v*/*v*)) and for CUPRAC 10 μL of the extracted sample was mixed with 190 μL of CUPRAC (10mM Cu(II); 7.5 mM neocuproine and 1 M acetate buffer pH 7; ratio 1:1:1 (*v*/*v*/*v*)). Both of them incubated the reaction mixture for 15 min at room temperature (25 ± 2 °C). Absorbance of the reaction mixture was measured at 630 nm for FRAP, and 450 nm for CUPRAC by using a BioTek ELX800 Absorbance Microplate Reader (BioTek Instruments).

### 2.9. Statistical Treatment of Data

The means and the standard deviation were used to present the data composed of three to six independent replicates. ANOVA was performed for comparative statistical analyses by using XLSTAT 2015 (Addinsoft, Paris, France). The correlation matrix was obtained with R corrplot package by performing the Pearson parametric correlation test to obtain a correlogram.

## 3. Results and Discussion

### 3.1. Single Laboratory Validation of a LC-Based Separation of the Silymarin Constituents

**Figure 2:**
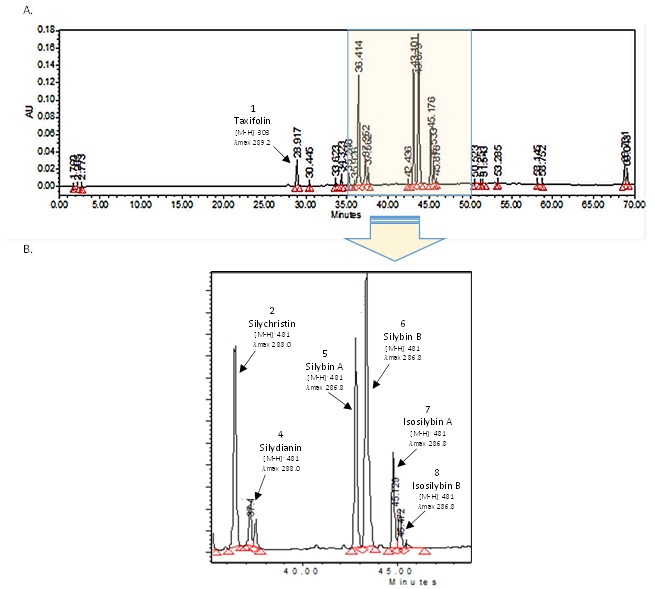
Representative chromatogram of a complete HPLC analysis (**A**). The main compounds considered in this study are the flavonoid taxifolin and all flavonolignans: silychristin, silydianin, silybin A, silybin B, isosilybin A, and isosilybin B. Presented on a magnification (**B**). Mass spectra are presented in Figure S1.

An optimal separation of the main constituents from a commercial silymarin mixture (Sigma Aldrich, S0292) was achieved using a core-shell column (Kinetex C18, 5 µm core-shell technology, Phenomenex) and a mobile phase that was consisting of a mixture of methanol and HPLC grade water, which were both acidified with 0.05% formic acid. In our hands, when compared to fully porous C18 column, the resolution was enhanced by the use of a core-shell column (data not shown). The superior efficiency and the improved peak symmetry and resolution of core-shell columns, when compared to the most common monolithic columns, for the analysis of the silybin (A and B) diastereoisomers were already described [22,23,41]. Moreover, the core-shell technology is enough versatile to allow for an easier possible transition of the present protocol to other methodologies, such as UPLC [41,42,43,44]. The typical LC-MS chromatogram (with detection set at 280 nm) is presented in Figure 2 and Figure S1. 

In Figure 2, the effective separation of taxifolin (28.92 min, *m*/*z* [M − H]^−^ 303, λ_max_ 289.2), silychristin (36.42 min, *m*/*z* [M − H]^−^ 481, λ_max_ 288.0), silydianin (7.65 min, *m*/*z* [M − H]^−^ 481, λ_max_ 288.0), silybin A (43.15 min, *m*/*z* [M − H]^−^ 481, λ_max_ 286.8), silybin B (43.29 min, *m*/*z* [M − H]^−^ 481, λ_max_ 288.0), isosilybin A (45.18 min, *m*/*z* [M − H]^−^ 481, λ_max_ 288.0), and isolsilybin B (45.89 min, *m*/*z* [M − H]^−^ 481, λ_max_ 288.0) is observed with a correct resolution of the method since all of the obtained Rs were greater than 1.5 (Table 1). Each compound was separated with a high stability, as indicated by their relative standard deviation in their retention time values ranging from 0.01, for silychristin, silybin A and isosilybin B, to 0.03 min for silydianin (i.e., lower than 0.01% variation). The detection and identification were asserted by both UV spectra and MS data as compared to those of authentic standards. To ensure precision and repeatability in the quantification, the method was then validated according to the association of analytical communities (AOAC) recommendations [45]. 

In our hands, the standard calibration curves exhibited correlation coefficient values that are greater than 0.9989 (Table 1). The limits of detection (LOD) and quantification (LOQ) ranged from 0.05 µg·mL^−1^ for silybins and isosilybins to 0.10 µg·mL^−1^ for silydianin for method LODs, and 0.15 µg·mL^−1^ for silybins to 0.32 µg·mL^−1^ for silydianin for method LOQs (Table 1). Our LODs and LOQs values are in the ranges that are described in the literature [41,44].

The repeatability precision was assessed by the percentage of relative standard deviation (RSD) as low as 5% and the Horwitz ratio (HorRat) as low as 1.69 (Table 2), which were calculated for the separation of each analyte from a standardized commercial Silymarin extract (S02092, Sigma Aldrich).

In order to evaluate the separation quality, the recovery of individual analytes was studied at three spiked concentration levels (Table 3).

The average recovery of each compound was at least 97.88% (for isosilybin A at 25 ppm spiked addition) and highest RSD as 7.71% (for isosilybin A at 50 ppm spiked addition) (Table 3), indicating a good stability of the separation. 

The matrix effect was also investigated using a 50 ppm concentration level addition on extracts from different origins: a commercial preparation of silymarin and an extract of laboratory-grown non-defatted milk thistle fruits obtained following ultrasonic extraction (Table 4).

Values of recovery that were close to 100% indicate that the origin of the extract does not affect the quality of the different separation constituents, thus highlighting the absence of the concentration and matrix effect (Table 4), and evidencing that the present separation method is quantitative.

**Figure 3:**
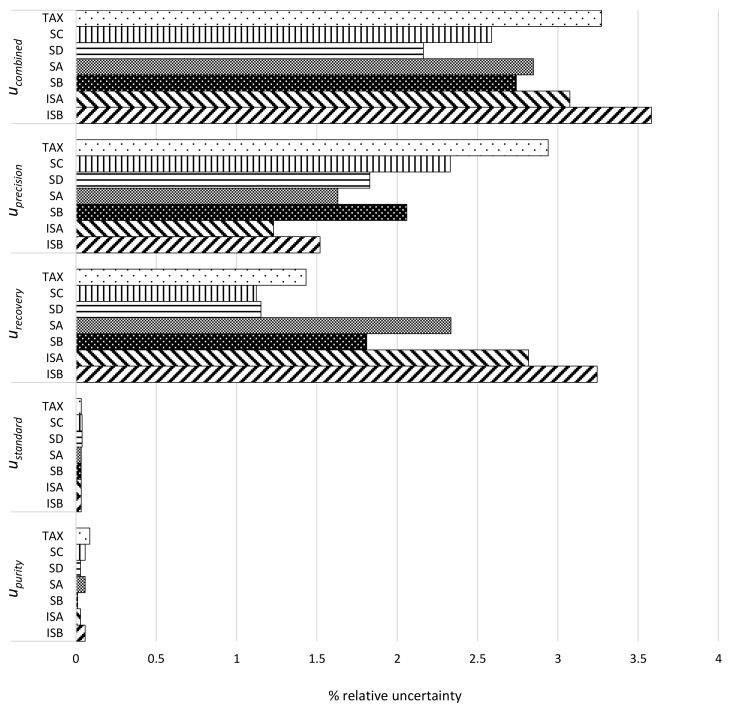
Uncertainty histogram showing the distribution of individual uncertainty component and combined expressed as percentage of taxifolin, silychristin, silydianin, silybin A, silybin B, isosilybin A, and isosilybin B in *S. marianum* preparation or extracts.

Uncertainty estimation of the final quantitative results was finally evaluated using standard a procedure that was described by Konieczka and Namiesnik [36], and the uncertainty calculations for analytical procedure devoted to natural antioxidants deriving from plant materials [34,35,36]. Sources of uncertainty were determined, as described in Figure S3. Uncertainties arising from standard stock purity u_purity_, standard preparation u_standard_, method recovery u_recovery_, and intermediate precision u_precision_ and combined uncertainty u_combined_ were calculated for each compound and were presented in the uncertainty histogram that is shown in Figure 3.

The combined uncertainty u_combined_ for the different analytes ranged from 2.16% for silydianin to 3.58% for isosilybin B (Figure 3). As expected, the main contributors to uncertainty were those that were resulting from method recovery and intermediate precision (Figure 3). These values are in the ranges that were described in the literature for methods applied to the quantification of natural antioxidants from plant materials [34,35]. According to the recommendations for accreditation and quality assurance [46], the obtained uncertainty values confirmed that the overall method performances provide an adequate estimation of the different silymarin constituents for practical applications. Expanded uncertainty (u_expanded_) using a coverage factor of 2 and at a 95% confidence level was estimated to be: ±7.2% for taxifolin, ±6.2% for silychristin, ±5.5% for silydianin, ±5.7% for silybin A, ±4.3% for silybin B, ±5.2% for isosilybin A, and ±6.5% for isosilybin B.

All together, these results confirmed that this single laboratory validated quantitative separation could be applied to the evaluation of silymarin constituents from different starting materials.

### 3.2. Evaluation of the Natural Variations in Silymarin Composition in Achene Extracts of Pakistani S. Marianum Accessions

Taking advantage of this validation separation method of the various constituents in silymarin mixture, its natural variation was further evaluated in the fruit (mature achene) of 12 wild Pakistani *Silybum marianum* samples (*P_Sm#*1-12). Non-defatted mature achenes were extracted using an ultrasound-assisted extraction procedure that is presented in Figure S4. This extraction procedure has been previously reported to enhance the amount of extracted silymarin extracted from mature fruits of *S. marianum* as compared to conventional methods, such as maceration [47]. Moreover, the defatting procedure has shown to result in severe silymarin loss [48]. With this extraction procedure, a huge variation in the silymarin content that ranged from 4.5 (*P_Sm*#10) to 23.6 (*P_Sm*#3) mg·g^−1^ DW of non-defatted whole fruits was observed (Table 5). This Silymarin variation range is comparable to those that were observed in other wild ecotypes from Egypt, Iran, Poland, Hungary, Bulgaria, Germany, Canada, and New Zealand (for review see [25]). Total phenolic and flavonoid contents were in the ranges of those that were previously reported [49]. This analysis revealed wild plants that contained high amounts of all these compounds (*P_Sm*#1 to #4), whereas some plants accessions were more specifically enriched in one compound: *P_Sm*#9 enriched in silybin A, *P_Sm*#5 and #7 enriched in silychristin, *P_Sm*#6 enriched in isosilybin or *P_Sm*#8 enriched in silydianin (Table 5). Concerning the individual variation in the different silymarin constituents, it ranged from: 0 to 2.1 mg·g^−1^ DW for taxifolin, 0.6 to 7.3 mg·g^−1^ DW for silychristin, 0.3 to 2.3 mg·g^−1^ DW for silydianin, 0.6 to 6.4 mg·g^−1^ DW for silybin A, 0.6 to 8.5 mg·g^-1^ DW for silybin B, 0 to 2.4 mg·g^−1^ DW for isosilybin A, and 0 to 2.3 mg·g^−1^ DW for isosilybin B (Table 5). These accumulation ranges were in line with those reported for other ecotypes [25].

It was mentioned that silymarin content and composition could deeply vary depending on the genetic background (i.e. ecotypes or cultivars) [30,50,51]. A strong fluctuation was also found in other studies, in particular, ecotypes from Egypt [52], Iran [50], as well as from Poland, Hungary, Bulgaria, and Germany [30]. 

**Figure 4:**
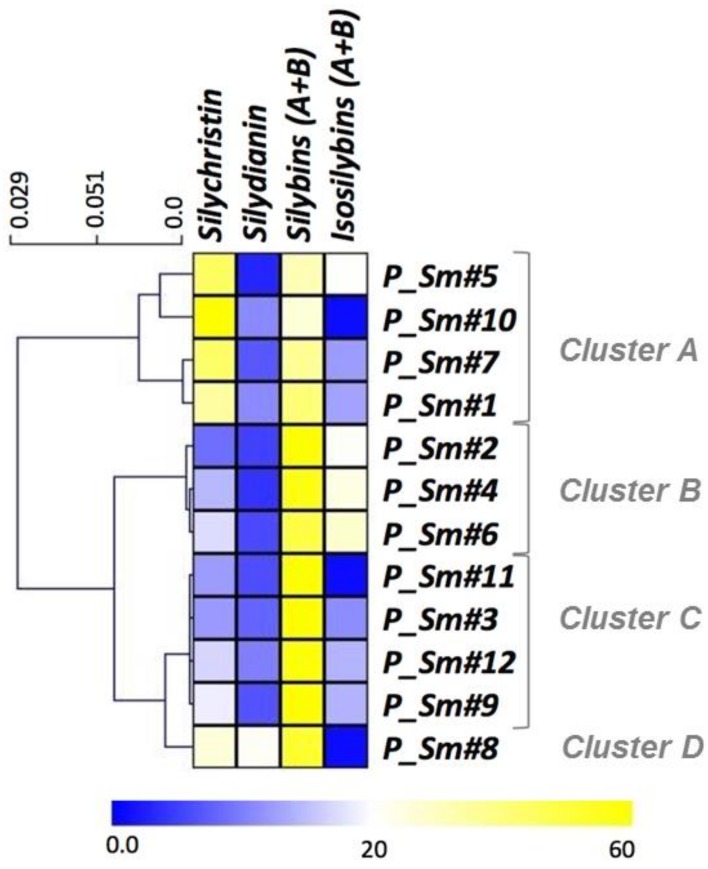
Hierarchical clustering analysis of twelve inflorescences of *Silybum marianum* related to their phytochemical profile.

The different ecotypes that were studied accumulated high levels of silybins (from 26% to 81% of total silymarin, for *P_Sm#10* and *P_Sm#11,* respectively). Five ecotypes accumulated around 60% of silybins (*P_Sm#2, #3, #9, #11* and *#12*) and four ecotypes of around 30% of silychristin (*P_Sm#1, #5, #7, #8,* and *#10*). Silydianin and isosilybins were accumulated in minor amounts: highest silydianin accumulations were around 20% for *P_Sm#8* and c.a. for *P_Sm#1, #10,* and *#12,* whereas the uppermost accumulations of isosilybins were around 20% for *P_Sm#2, #4, #5,* and *#6,* and close to 10% for *P_Sm#1, #3, #7, #9,* and *#12*. All of these trends were presented in a heatmap representation and were analyzed through a hierarchical clustering analysis (HCA) in order to identify the potential chemotypes (Figure 4). 

It has been previously shown that the composition of silymarin mixture depends on the *Silybum marianum* ecotypes from different countries [30]. Based on their phytochemical diversity, some of these milk thistles have been considered as chemotypes. To date, only three distinct *Silybum marianum* chemotypes have been described based on their silymarin composition: chemotype A, accumulating around 30% of silychristin and 60% of silybins; chemotype B, showing a silydianin content that was exceeding 70%; and, chemotype C, which was considered as an intermediate between chemotypes A and B with approximately 17% of silychristin, 40% silydianin and 30% silybins [53]. In our hands, *P_Sm#1, #5, #7* and *#10* from cluster A clearly matched the composition defined for chemotype A. As reported for chemotype C [53], *P_Sm#8* presented a more balanced composition of silychristin, silydianin, and silybins, but without perfectly matching the percentages that were defined for this chemotype and could therefore be addressed to an enlarged chemotype C. The chemical profile of the two last clusters B and C, here observed, were not described to date and therefore constitute new chemotypes: chemotype D composed of 50% silybins and 20% isosilybins for cluster B grouping accessions *P_Sm#2, #4,* and *#6 and* chemotype E with a prominent accumulation of silybins (more than 60%) for cluster C grouping accessions *P_Sm#3, #9, #11,* and *#12.* Note that the silydianin-rich genotypes that were first described by Adzet et al. [54] were not found here within the 12 analyzed ecotypes. Further analyses will be necessary to conclude on the occurrence/absence of such silydianin-rich chemotype among Pakistani ecotypes.

**Figure 5:**
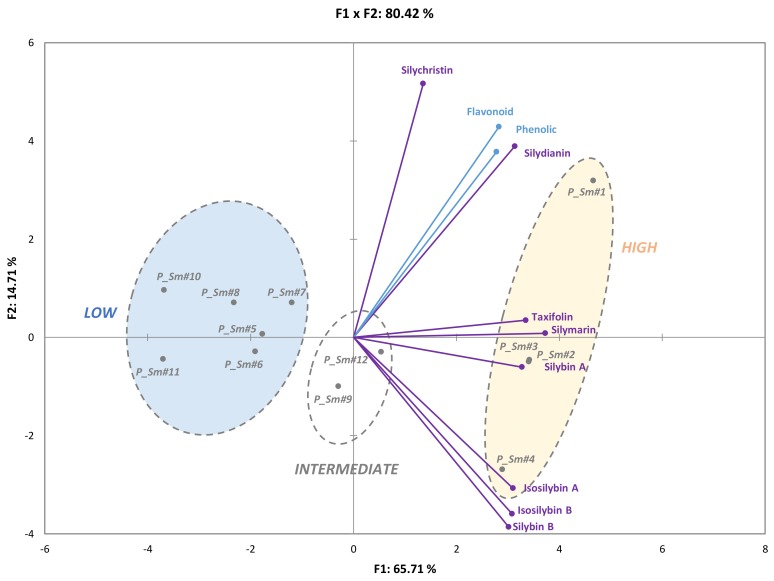
Principal component analysis (PCA) of the different silymarin compounds and its extraction from twelve inflorescences. Variance of factor 1 (F1) = 65.71% and of factor 2 (F2) = 14.71%. Ecotypes are distributed into the three groups (Low, intermediate and high) according to their amount of extraction.

Chemotypes could give important information about the biosynthesis sequence leading to the different flavonolignans constituting of the complex silymarin mixture. Despite the pharmacological and economic importance of these derivatives, to date, little is known about this biosynthetic pathway and its regulation. A principal component analysis (PCA) allow for us to further discriminate these 12 samples. The resulting biplot representation accounts for 80.42% (F1 + F2) of the initial variability of the data (Figure 5). Discrimination occurs mainly in the first dimension (F1 axis) which explains 65.71% of the initial variability and silymarin content, together with silybin A and taxifolin contents, was the main contributor for this axis. The other phytochemicals that were studied appeared to contribute to the second dimension (F2 axis), accounting for only 14.71% of the initial variability (Figure 2). PCA showed a significant grouping of samples as a function of their silymarin content. Using this analysis, the different ecotypes could also be easily discriminated into three different clusters depending on their phytochemical profile (Figure 4).This observation could be related to genetic data available for Pakistani genotypes [55]. The first cluster of four ecotypes (*P_Sm#1, #2, #3,* and *#4*) grouped ecotype together with high SM amount (high; Figure 4), as well as strong antioxidant activities (Figure 5). Contrarily, the last group is composed of six ecotypes (*P_Sm*#5, #6, #7, #8, #10, and #11) with low SM levels (low; Figure 5). The two remaining ecotypes (*P_Sm*#9 and *#12*) constitute the second cluster with an intermediate flavonolignans quantities and antioxidant activities (intermediate; Figure 5).

These results reflect the great natural genetic variability of these populations in terms of flavonolignans bioaccumulation. A similar separation was also proposed in a study of both wild and commercial populations from Iran, demonstrating the richness of wild ecotypes that will deserve further experiments [56]. Here, the wild ecotypes originating from Pakistan constitutes a natural source of biodiversity, moreover, due to the lack of cultivation of commercial varieties in this region, the risk of analyzing escape from commercial populations is lower. These 12 ecotypes showed chemical variations of their secondary metabolites due to the influence of their genetic background, although their morphology is not substantially transformed, only their chemical phenotype is shifting. 

### 3.3. Correlations between Phytochemical Profiles and Antioxidant Activities

**Figure 6:**
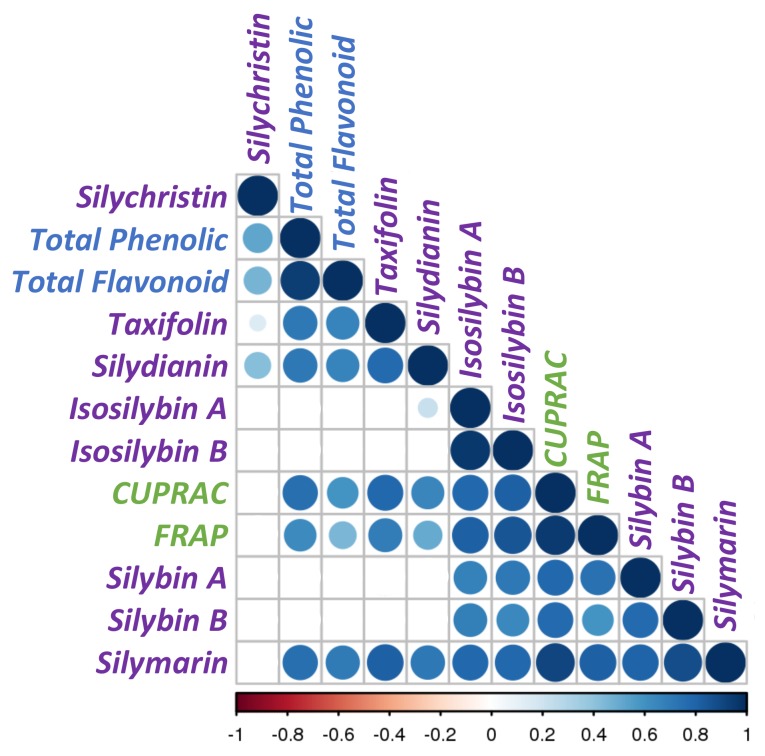
Correlogram analysis between phytochemical profiles of *Silybum marianum* extracts analyzed in this study and their antioxidant activities. Positive correlations are displayed in blue and negative correlations in red. Color intensity and the size of the circles are proportional to the correlation coefficients. Below the correlogram of flavonolignans and antioxidant tests, the legend color shows the correlation coefficients and the corresponding colors.

To evaluate the linkage between the antioxidant activity and the phytochemical profile of these chemotypes, Pearson correlation coefficient (PCC) were calculated and a matrix was constructed (Figure 6). This analysis evidenced the strength of the relationship between several variables (Figure 6 and Table S1). Not surprisingly, this correlogram showed a strong and highly significant (*p* < 0.001) correlation between silymarin content and the in vitro antioxidant assays CUPRAC (PCC = 0.92) and FRAP (PCC = 0.91). Total phenolic acids and total flavonoid contents were also significantly correlated with these two antioxidant assays, but with a weaker strength, suggesting that silymarin is the main contributor for the antioxidant capacity of these *Silybum marianum* extracts. With the exception of silychristin, all of the other constituents of the silymarin mixture were significantly correlated with CUPRAC and FRAP assays. We note that the PCCs that were calculated between each of the different constituents of the silymarin mixture and the antioxidant assays did not reach the value that was obtained for the complete mixture, indicating a possible synergistic effect. A stronger correlation (higher PCCs and more confident *p*-values) was observed with the CUPRAC assay for taxifolin, silydianin, as well as total phenolics and flavonoids, whereas both diasteroisomers of silybin and isosilybin were strongly correlated with the FRAP assay. When considering that the FRAP assay evidences the antioxidant activity of hydrophilic substances, whereas CUPRAC assay is more appropriate for both lipophilic and hydrophilic antioxidants [57], the two distinct behaviors merely relied on the physical and chemical properties of these compounds.

Little is known about the biosynthetic pathway leading to the flavonolignan biosynthesis. Our current knowledge on the flavonolignan biosynthesis involved the one-electron oxidation of two precursors (*E*-coniferyl alcohol and taxifolin), followed by a random coupling of those radicals, leading to the formation of one or several quinone methide intermediate(s) to form the different silymarin flavonolignans. With such a mechanism, only equimolar mixtures silybin (A and B) and isosilybin (A and B) diasteroisomers are supposed to be produced. However, some authors have reported different molar ratios [31,51,58,59]. Recently, Martinelli et al. [53] suggested the possible involvement of dirigent-like proteins that could explain such a discrepancy. Dirigent proteins (from the Latin: *dirigere*, to guide or to align) were first described to guide the stereoselective coupling of two phenoxy radicals that were derived from *E*-coniferyl alcohol, to give (+)-pinoresinol provided one electron oxidative capacity was also present to generate the phenoxy radical species [60]. To date there is no consensus about radical coupling mechanism involved in the biosynthesis of silybin and isosilybin diastereoisomers from *Silybum marianum*. Correlation analysis could also reveal the biochemical connectivity between the different intermediates and/or the branch of a biosynthetic pathway, as demonstrated by Nguyen et al. [61]. These authors proposed that the absence of correlation between substrate and the product of a considered enzymatic conversion suggests a strong contribution of this conversion to the flux control of the biosynthetic pathway and therefore a more complex regulation [61]. Such an enzymatic conversion is therefore a good candidate target for metabolic engineering. Here, PCCs suggested that silydianin and silychristin biosynthesis on the one hand, and silybins and isosilybins on the other hand, are governed by distinct control mechanisms. Indeed, strong and highly significant correlations linked taxifolin to silychristin and silychristin, whereas no correlations linked taxifolin to silybins, and isosilybins (Figure 6, Table S2). These results could therefore suppose a different mechanism leading to the biosynthesis of these flavonolignans involving random radical coupling for the biosynthesis of silychristin and silydianin, and the involvement of a dirigent-like protein for the biosynthesis of silybins and isosilybins. Nevertheless, in our hands, except *P_Sm#4*, the molar ratio of the different diastereoisomers were close to the 1:1 ratio that was observed in the case of random coupling, but the involvement of a dirigent-like protein cannot be excluded. Indeed, dirigent proteins with opposite stereospecificities could be involved, as observed for the biosynthesis of lignans in flax seeds [62]. These *S. marianum* ecotypes can be a good material to determine the biosynthetic relation linking the different flavonolignans and their precursor taxifolin, and to hypothesize the enzymatic pathways that are involved in their biosynthesis. 

## 4. Conclusions

An efficient quantitative separation method for the concentration level evaluation of the different flavonolignans of the silymarin mixture from complex material have been validated. This method was applied to the phytochemical characterization of 12 wild *Silybum marianum* accessions from Pakistan and evidenced their pronounced chemical variability. Among these accessions, two new chemotypes that are rich in silybins and isosilybins are here described for the first time. A possible synergistic effect between the different constituents of the silymarin mixture to reach maximal antioxidant potential is proposed. Correlation analysis also suggested distinct control mechanisms leading to the biosynthesis of these flavonolignans, such as a random radical coupling for the biosynthesis of silychristin and silydianin, and the possible action of dirigent-like protein for the biosynthesis of silybin and isosilybin diastereoisomers. When considering that silybin is the most bioactive component of the silymarin mixture, the two newly described chemotypes are of particular interest and could have a great potential for cosmetic and pharmaceutical applications.

## Figures and Tables

**Table 1:** Calibration function parameters for the different analytes using UV detection.

Analyte	Retention Time *t*_R_			Calibration Curve			LOD	LOQ
	min	RSD	R_S_	slope	intercept	R^2^	(µg·mL^−1^)	(µg·mL^−1^)
Taxifolin	28.92	0.02	6.65	1292.9	0.7	0.9989	0.09	0.26
Silychristin	36.42	0.01	5.58	2266.4	−12.7	0.9994	0.05	0.17
Silydianin	37.65	0.03	1.71	1649.8	34.8	0.9992	0.10	0.32
Silybin A	43.15	0.01	1.69	2575.0	5.7	0.9997	0.05	0.15
Silybin B	43.29	0.02	1.69	2515.9	27.3	0.9999	0.05	0.15
Isosilybin A	45.18	0.02	1.78	2726.3	17.8	0.9998	0.05	0.16
Isosilybin B	45.89	0.01	1.78	2861.1	1.9	0.9999	0.05	0.16

Linear range: 0.5–50 µg·mL^−1^; *t*_R_: retention time; RSD: Relative Standard deviation; R_S_: separation resolution; LOD: limit of detection; LOQ: limit of quantification

**Table 2:** Repeatability precision results summary for the quantification of the different analytes in standardized commercial silymarin preparation from milk thistle.

Analyte	Mean (mg·g^−1^)	RSD (%)	HorRat
Taxifolin	2.46 ± 0.10	4.17	1.69
Silychristin	13.31 ± 0.42	3.16	1.64
Silydianin	1.85 ± 0.05	2.60	1.01
Silybin A	10.59 ± 0.24	2.31	1.17
Silybin B	8.42 ± 0.24	2.92	1.43
Isosilybin A	5.01 ± 0.09	1.75	0.78
Isosilybin B	2.31 ± 0.05	2.15	1.69

Values are presented means of 6 independent replicates (mg·g^−1^), percentage of relative standard deviation (RSD) and Horwitz ratio (HorRat) for each analyte.

**Table 3:** Recovery results for individual analytes at three spiked concentrations in negative control (extraction solvent).

Analyte	Spiked Concentration (ppm)	Recovery (%)	Average Recovery (%)
Taxifolin	25	101.85 ± 1.98	
	50	102.77 ± 3.80	
	100	100.27 ± 0.35	101.63 ± 2.04
Silychristin	25	102.25 ± 3.33	
	50	99.70 ± 0.26	
	100	99.96 ± 0.45	100.64 ± 1.35
Silydianin	25	100.52 ± 3.10	
	50	100.78 ± 0.46	
	100	98.41 ± 1.46	99.90 ± 1.67
Silybin A	25	101.75 ± 4.67	
	50	102.45 ± 5.10	
	100	99.00 ± 1.12	101.07 ± 3.63
Silybin B	25	100.66 ± 3.84	
	50	98.09 ± 2.55	
	100	98.89 ± 2.87	99.22 ± 3.09
Isosilybin A	25	97.88 ± 2.16	
	50	100.06 ± 7.71	
	100	101.54 ± 2.72	99.83 ± 4.20
Isosilybin B	25	102.17 ± 6.79	
	50	102.93 ± 5.25	
	100	101.89 ± 4.60	102.33 ± 5.55

Values are presented as recovery percentage means of 3 independent replicates (recovery) ± relative standard deviation (RSD) and average of recovery obtained for the three spiked concentrations.

**Table 4:** Estimation of matrix effects by recovery results for individual analytes at spiked concentration of 50 ppm in two different matrixes.

Matrix	Taxifolin	Silychristin	Silydianin	Silybin A	Silybin B	Isosilybin A	Isosilybin B
Silymarin extract ^a^	98.91 ± 2.04	99.54 ± 2.06	94.42 ± 4.65	100.77 ± 1.53	100.26 ± 1.18	100.33 ± 1.95	101.96 ± 3.21
Milk thistle seed extract ^b^	99.60 ± 2.24	99.15 ± 0.04	92.00 ± 1.68	99.48 ± 0.81	98.99 ± 1.48	98.22 ± 1.22	100.53 ± 4.77

Values (*n* = 3) are the percent mean of the spiked analyte recovery ± %RSD; Values close to 100 indicate no matrix effect observed; ^a^ Standardized commercial Silymarin preparation from Sigma Aldrich (reference S0292); ^b^ non-defatted whole seed extract prepared as described in Materials and Methods.

**Table 5:** Content of silymarin constituents, total phenolics, total flavonoids, and antioxidant capacity of different genotypes of milk thistle from Pakistan.

Sample	TAX^a^	SC^a^	SD^a^	SA^a^	SB^a^	ISA^a^	ISB^a^	SM^b^	Phe^c^	Fla^d^	CUPRAC^e^	FRAP^e^
*P_Sm#1*	2.0 ±0.3^AB^	7.3 ±1.9^ABC^	2.3 ±0.2^A^	4.9 ±0.2^AB^	3.8 ±0.4^ABC^	1.4 ±0.0^B^	1.3 ±0.0^A^	22.9 ±2.2^AB^	363.6 ±35.9^A^	51.4 ±5.3^A^	1257.1 ±162.2^A^	1657.9 ±91.2^AB^
*P_Sm#2*	2.1 ±0.3^AB^	1.7 ±0.1^ABC^	1.0 ±0.2^ABCD^	6.6 ±0.1^A^	6.0 ±0.2^ABC^	2.3 ±0.2^A^	1.8 ±0.0^A^	21.5 ±0.7^AB^	247.0 ±5.5^AB^	45.5 ±2.4^A^	995.2 ±50.6^AB^	1375.8 ±218.8^AB^
*P_Sm#3*	2.1 ±0.2^A^	2.6 ±0.2^ABC^	1.7 ±0.1^AB^	6.4 ±0.4^AB^	8.5 ±0.3^A^	1.1 ±0.0^B^	1.3 ±0.0^AB^	23.6 ±0.1^A^	197.6 ±11.7^AB^	24. ±4.6^AB^	1165.8 ±40.9^A^	1509.3 ±53.6^AB^
*P_Sm#4*	1.4 ±0.2^ABC^	2.8 ±0.2^ABC^	0.8 ±0.2^ABCD^	3.5 ±0.1^BCDE^	7.5 ±0.8^AB^	2.4 ±0.1^A^	2.3 ±0.1^A^	20.7 ±0.9^AB^	121.2 ±13.1^ABC^	14.0 ±1.1^B^	1237.7 ±79.5^A^	1843.3 ±74.9^A^
*P_Sm#5*	nd ^D^	4.6 ±0.2^AB^	0.3 ±0.0^D^	1.5 ±0.1^DEFG^	1.7 ±0.0^DE^	1.0 ±0.0^B^	1.1 ±0.0^AB^	10.2 ±0.3^DEF^	128.8 ±11.5^ABC^	16.0 ±1.2^AB^	731.0 ±44.7^AB^	1235.8 ±138.8^AB^
*P_Sm#6*	1.2 ±0.1^ABC^	1.8 ±0.3^ABC^	0.6 ±0.3^ABC^	1.8 ±0.1^CDEF^	3.3 ±0.3^BCDE^	1.3 ±0.0^B^	1.6 ±0.1^A^	11.6 ±0.9^CDE^	137.1 ±9.9^ABC^	16.6 ±3.0^AB^	866.4 ±36.3^AB^	1357.8 ±77.2^AB^
*P_Sm#7*	0.8 ±0.1^BCD^	5.7 ±0.2^A^	0.9 ±0.2^ABCD^	1.4 ±0.0^EFG^	3.4 ±0.2^ABCDE^	0.8 ±0.0^B^	0.8 ±0.0^AB^	13.8 ±0.3^CD^	81.3 ±28.7^C^	19.0 ±0.6^AB^	636.7 ±150.4^B^	1120.0 ±259.1^AB^
*P_Sm#8*	1.4 ±0.1^ABC^	1.6 ±0.1^BC^	1.3 ±0.3^ABC^	0.6 ±0.0^G^	2.6 ±0.1^CDE^	nd ^C^	nd ^B^	7.6 ±0.4^CDEF^	86.9 ±9.4^C^	15.1 ±2.7^AB^	623.2 ±92.7^B^	1012.3 ±150.4^B^
*P_Sm#9*	0.1 ±0.0^CD^	2.0 ±0.4^ABC^	0.7 ±0.0^ABCD^	3.8 ±0.2^ABC^	2.7 ±0.3^CDE^	0.8 ±0.0^B^	0.7 ±0.0^AB^	11.0 ±0.2^CDEF^	82.3 ±15.6^C^	16.1 ±1.0^AB^	702.5 ±42.2^AB^	975.6 ±220.1^AB^
*P_Sm#10*	nd ^D^	2.9 ±0.1^ABC^	0.5 ±0.0^BCD^	0.6 ±0.0^G^	0.6 ±0.1^E^	nd ^C^	nd ^B^	4.5 ±0.3^F^	105.1 ±11.3^BC^	16.5 ±1.1^AB^	587.0 ±16.3^B^	994.7 ±61.1^B^
*P_Sm#11*	nd ^D^	0.6 ±0.0^C^	0.3 ±0.1^CD^	1.2 ±0.1^FG^	2.8 ±0.2^CDE^	nd ^C^	nd ^B^	4.9 ±0.1^EF^	64.3 ±15.2^C^	12.6 ±2.1^B^	580.4 ±57.9^B^	1041.0 ±53.5^B^
*P_Sm#12*	0.9 ±0.2^ABCD^	2.5 ±0.0^ABC^	1.5 ±0.2^AB^	3.7 ±0.3^ABCD^	5.1 ±0.5^ABC^	0.9 ±0.0^B^	1.2 ±0.1^AB^	15.8 ±0.7^ABC^	122.2 ±23.3^ABC^	20.3 ±3.8^AB^	878.0 ±112.8^AB^	1327.8 ±102.9^AB^

^a^ quantified by HPLC and expressed in mg·g^−1^ DW; ^b^ expressed in as the sum of in mg·g^−1^ DW taxifolin and flavonolignans quantified by HPLC; ^c^ Total phenolic estimated by Folin assay expressed in mg·g^−1^ DW gallic acid equivalent; ^d^ Total flavonoid content estimated by AlCl_3_ assay and expressed in mg·g^−1^ DW quercetin equivalent; ^e^ expressed in µM of Trolox equivalent antioxidant capacity. Each value represented mean ± SD of three independent replicate; nd non-determined (under the limits of quantification (LOQ)) for taxifolin and non-detectable (under the limits of detection (LOD) for isosilybin A and isosilybin B; superscript letters indicate differences on ANOVA at α = 0.05).

## Supplementary Materials

The following are available online. Figure S1: MS spectra of the main flavonolignans from *S. marianum* extract: silychristin, silydianin, silybin A, silybin B, isosilybin A and isosilybin B, Figure S2: Identification of possible experimental errors present in the analysis, Figure S3: Analytical methodology for the extraction and analysis of the main flavonolignans from *S. marianum* extract, Table S1: Pearson Correlation Matrix for relationships between flavonolignan, total phenolic and total flavonoid contents and in vitro antioxidant activities.
