# Supplementary material for: Single Laboratory Validation of a Quantitative Core Shell-Based LC Separation for the Evaluation of Silymarin Variability and Associated Antioxidant Activity of Pakistani Ecotypes of Milk Thistle (Silybum Marianum L.)
Source: Molecules. 2018 Apr 14;23(4):904. doi: 10.3390/molecules23040904 (PMC6017045; doi:10.3390/molecules23040904)
Supplement: Supplementary file 1 [file molecules-23-00904-s001.pdf]

molecules

Supplementary Materials

Single laboratory validation of a quantitative core shell-based LC separation for the evaluation of silymarin variability and antioxidant activity of Pakistani ecotypes of milk thistle (*Silybum marianum* L.) achene extracts

Samantha Drouet[12], Bilal Haider Abbasi[3], Annie Falguières[4], Waqar Ahmad[3], Sumaira[3], Clothilde Ferroud[4], Joël Doussot[124], Jean Raymond Vanier[6], Eric Lainé[12], Christophe Hano[12*]

[1] Laboratoire de Biologie des Ligneux et des Grandes Cultures (LBLGC), INRA USC1328, Université d'Orléans, Pôle Universitaire d'Eure et Loir, 21 rue de Loigny la Bataille, 28000 Chartres, France; samantha.drouet@univ-orleans.fr; eric.laine@univ-orleans.fr; hano@univ-orleans.fr;
[2] Bioactifs et Cosmétiques, GDR 3711 COSMACTIFS, CNRS/Université d'Orléans, France;
[3] Department of Biotechnology, Quaid-i-Azam University, Islamabad 45320, Pakistan; bhabbasi@qau.edu.pk; awaqar@bs.qau.edu.pk; sumaira.khan1890@gmail.com;
[4] Ecole Sciences industrielles et technologiques de l'information (SITI), Département Chimie Alimentation Santé Environnement Risque (CASER), Le CNAM Paris, France; annie.falguieres@cnam.fr; joel.doussot@cnam.fr; clothilde.ferroud@cnam.fr;
[5] Plantes Médicinales et Aromatiques 28, PMA28, 1, place de l'Eglise, 28140 Varize, France; jraymond.vanier@pma28.fr;

* Correspondence: hano@univ-orleans.fr; Tel.: +33 2 37 30 97 53

Table S1. Pearson Correlation Matrix for relationships between flavonolignans, antioxidant activities, phenolic and flavonoid test.

Variables	Phenolic	Flavonoid	Taxifolin	Silychristin	Silydianin	SilybinA	SilybinB	IsosilybinA	IsosilybinB	Silymarin	CUPRAC	FRAP
**Phenolic**												
**Flavonoid**	**0.94***											
**Taxifolin**	**0.92***	**0.91***										
**Silychristin**	**0.77**	**0.71**	**0.60***									
**Silydianin**	**0.79**	**0..75**	**0.85***	**0.65***								
**SilybinA**	0.20	0.14	0.49	-0.18	0.49							
**SilybinB**	0.29	0.23	0.54	-0.08	0.57	**0.97***						
**IsosilybinA**	0.47	0.34	0.55	0.10	**0.59***	**0.73**	**0.80**					
**IsosilybinB**	0.40	0.26	0.43	0.15	0.46	**0.63***	**0.69***	**0.92***				
**Silymarin**	**0.69***	**0.60***	**0.84**	0.42	**0.86***	**0.80**	**0.86***	**0.79**	**0.70***			
**CUPRAC**	**0.78**	**0.73**	**0.89***	0.37	**0.80**	**0.70***	**0.77**	**0.85**	**0.74**	**0.92***		
**FRAP**	**0.70***	**0.64***	**0.83**	0.32	**0.70***	**0.75**	**0.8**	**0.85**	**0.79**	**0.91***	**0.97***	

* (p<0.05), ** (p<0.01) and *** (p<0.001)

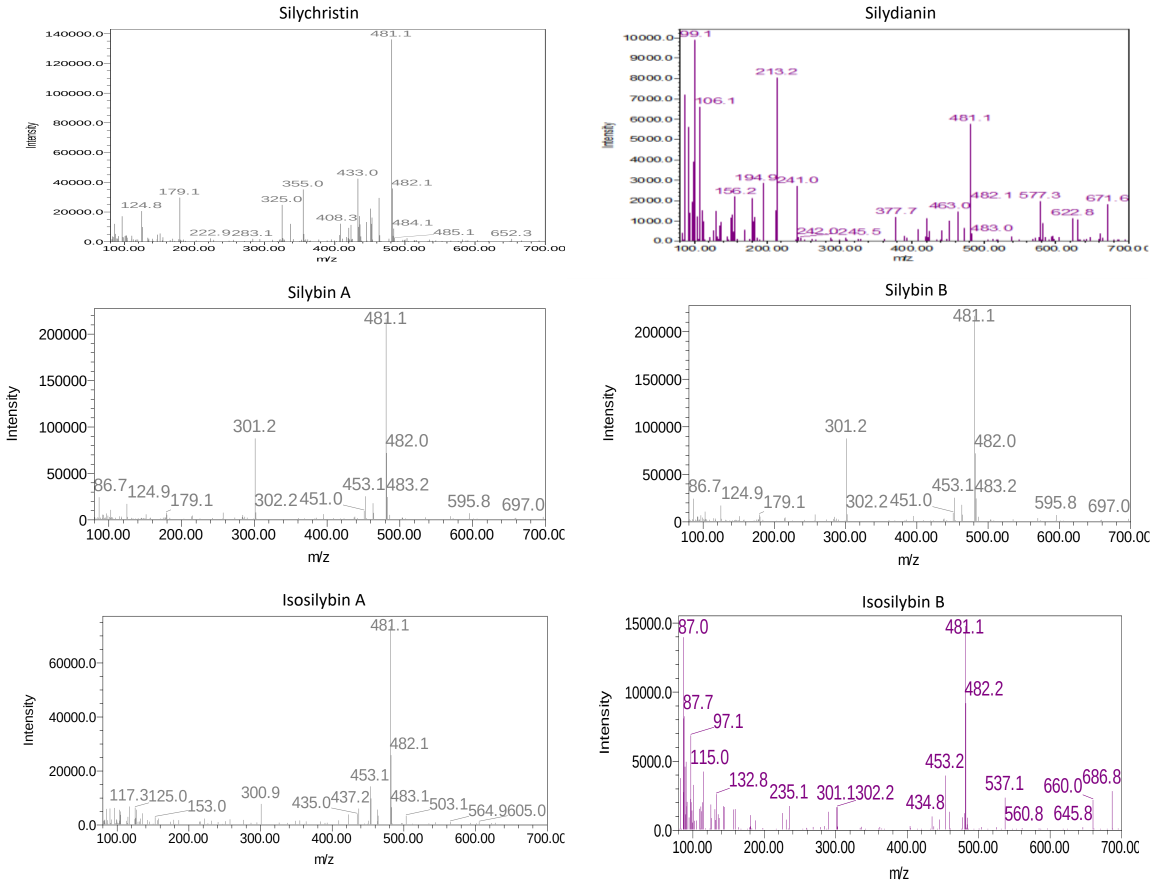

Figure S1. MS spectra of the main flavonolignans from *S. marianum* extract: silychristin, silydianin, silybin A, silybin B, isosilybin A and isosilybin B.

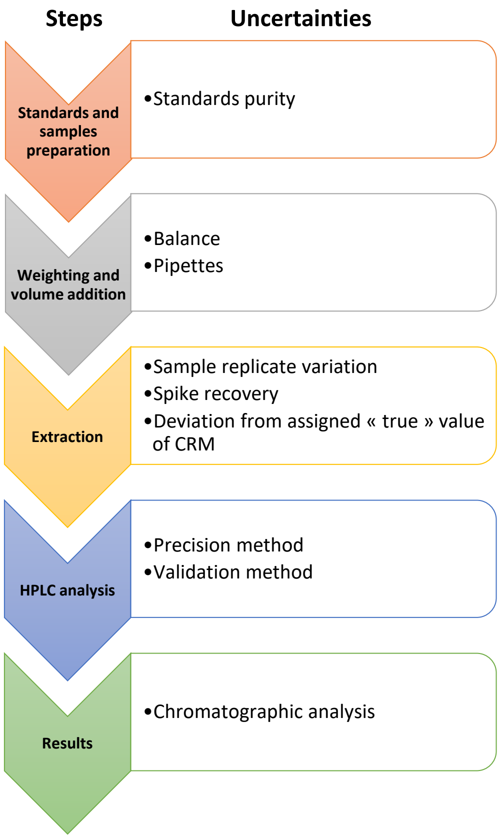

Figure S2. Identification of possible experimental errors present in the analysis.

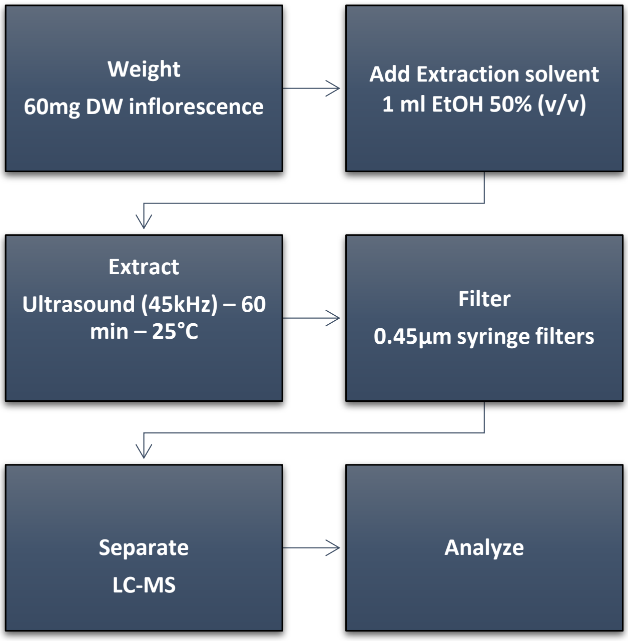

Figure S3. Analytical methodology for the extraction and analysis of the main flavonolignans from *S. marianum* extract.
